# An SIRS Epidemic Model Incorporating Media Coverage with Time Delay

**DOI:** 10.1155/2014/680743

**Published:** 2014-03-03

**Authors:** Huitao Zhao, Yiping Lin, Yunxian Dai

**Affiliations:** ^1^Department of Applied Mathematics, Kunming University of Science and Technology, Kunming, Yunnan 650093, China; ^2^Department of Mathematics and Information Science, Zhoukou Normal University, Zhoukou, Henan 466001, China

## Abstract

An SIRS epidemic model incorporating media coverage with time delay is proposed. The positivity and boundedness are studied firstly. The locally asymptotical stability of the disease-free equilibrium and endemic equilibrium is studied in succession. And then, the conditions on which periodic orbits bifurcate are given. Furthermore, we show that the local Hopf bifurcation implies the global Hopf bifurcation after the second critical value of the delay. The obtained results show that the time delay in media coverage can not affect the stability of the disease-free equilibrium when the basic reproduction number *R*_0_ < 1. However, when *R*_0_ > 1, the stability of the endemic equilibrium will be affected by the time delay; there will be a family of periodic orbits bifurcating from the endemic equilibrium when the time delay increases through a critical value. Finally, some examples for numerical simulations are also included.

## 1. Introduction

Since Kermack and Mckendrick proposed the classical SIR epidemic model in 1927, mathematical modeling has become important tools in analyzing the spread and control of infectious diseases. Attempts have been made to develop realistic mathematical models for the transmission dynamics of infectious diseases. In recent years, epidemic models described by ordinary differential equations have been studied by many authors (see, e.g., [1–9] and the references cited therein).

One of the most fundamental compartment models based on differential equations is the SIRS model described by (1) below [10–15]. Let *S*(*t*) be the number of susceptible individuals, *I*(*t*) the number of infective individuals, and *R*(*t*) the number of removed individuals at time *t*, respectively. A general SIRS epidemic model can be formulated as

(1) $$\begin{array}{c} \frac{dS}{dt}=b-dS-g\left(I\right)S+\gamma R,\\ \frac{dI}{dt}=g\left(I\right)S-\left(d+\mu +\delta \right)I,\\ \frac{dR}{dt}=\mu I-\left(d+\gamma \right)R, \end{array}$$

where *b* > 0 is the recruitment rate of the population, *d* > 0 is the natural death rate of the population, *μ* > 0 is the natural recovery rate of the infective individuals, *γ* > 0 is the rate at which recovered individuals lose immunity and return to the susceptible class, and *δ* > 0 is the disease-induced death rate. The transmission of the infection is governed by the incidence rate *g*(*I*)*S*, and *g*(*I*) is called the infection force.

In modelling of communicable diseases, the incidence rate *g*(*I*)*S* may be affected by some factors, such as media coverage, density of population, and life style [16–22]. It is worthy to note that media coverage plays an important role in helping both the government authority make interventions to contain the disease and people respond to the disease [16, 19]. And a number of mathematical models have been formulated to describe the impact of media coverage on the transmission dynamics of infectious diseases. In particular, Cui et al. [16], Tchuenche et al. [18], and Sun et al. [20] incorporated a nonlinear function of the number of infective individuals (2) in their transmission term to investigate the effects of media coverage on the transmission dynamics:

(2) $$\begin{array}{c} g\left(I\right)=\beta_{1}-\frac{\beta_{2}I}{m+I}, \end{array}$$

where *β*_1_ > 0 is the maximal effective contact rate between the susceptible and infective individuals and *β*_2_ > 0 is the maximal reduced effective contact rate due to mass media alert in the presence of infective individuals; the terms *β*_2_ *I*/(*m* + *I*) measure the effect of reduction of the contact rate when infectious individuals are reported in the media. Because the coverage report cannot prevent disease from spreading completely we have *β*_1_⩾*β*_2_ > 0. The half-saturation constant *m* > 0 reflects the impact of media coverage on the contact transmission. The function *I*/(*m* + *I*) is a continuous bounded function which takes into account disease saturation or psychological effects [20–22]. Then model (1) becomes

(3) $$\begin{array}{c} \frac{dS}{dt}=b-dS-\left(\beta_{1}-\frac{\beta_{2}I}{m+I}\right)SI+\gamma R,\\ \frac{dI}{dt}=\left(\beta_{1}-\frac{\beta_{2}I}{m+I}\right)SI-\left(d+\mu +\delta \right)I,\\ \frac{dR}{dt}=\mu I-\left(d+\gamma \right)R. \end{array}$$

On the other hand, delays are ubiquitous in life, so it is in media coverage. Media coverage of an infectious outbreak can be seen as following two major routes [20, 23]. The first route is when the media report directly to the public on facts that they (the media) observe; the second has public health authorities using mass media or the Internet to communicate about the outbreak. For the second route, the number of infections and the number of suspected infections reported by media today are often the statistical result of yesterday or the day before. So the effects of media coverage on the transmission dynamics can be modified as follows:

(4) $$\begin{array}{c} g\left(I\left(t-\tau \right)\right)=\beta_{1}-\frac{\beta_{2}I\left(t-\tau \right)}{m+I\left(t-\tau \right)}, \end{array}$$

where *τ* > 0 is a time delay representing the latent period of media coverage. Then model (3) can be modified as

(5) $$\begin{array}{c} \frac{dS\left(t\right)}{dt}=b-dS\left(t\right)-\left(\beta_{1}-\frac{\beta_{2}I\left(t-\tau \right)}{m+I\left(t-\tau \right)}\right)S\left(t\right)I\left(t\right)+\gamma R\left(t\right),\\ \frac{dI\left(t\right)}{dt}=\left(\beta_{1}-\frac{\beta_{2}I\left(t-\tau \right)}{m+I\left(t-\tau \right)}\right)S\left(t\right)I\left(t\right)-\left(d+\mu +\delta \right)I\left(t\right),\\ \frac{dR\left(t\right)}{dt}=\mu I\left(t\right)-\left(d+\gamma \right)R\left(t\right). \end{array}$$

In the following, we will investigate the effect of time delay on the dynamics of system (5). We suppose that the initial condition for system (5) takes the form

(6) S(θ)=ϕ1(θ),  I(θ)=ϕ2(θ),  R(θ)=ϕ3(θ),    ϕi(θ)≥0, θ∈[−τ,0], ϕi(0)>0,                   i=1,2,3,

where (*ϕ*_1_(*θ*), *ϕ*_2_(*θ*), *ϕ*_3_(*θ*)) ∈ *𝒞*([−*τ*, 0], **R**_+0_^3^), which is the Banach space of continuous functions mapping the interval [−*τ*, 0] into **R**_+0_^3^, where **R**_+0_^3^ = {(*x*, *y*, *z*) | *x* ≥ 0, *y* ≥ 0, *z* ≥ 0}.

By the fundamental theory of functional differential equations [24], system (5) has a unique solution (*S*(*t*), *I*(*t*), *R*(*t*)) satisfying the initial condition (6).

The rest of the paper is organized as follows. In Section 2, we show the positivity and the boundedness of solutions of system (5) with initial condition (6). In Section 3, we study the local stability of the equilibria and the existence of the Hopf bifurcation at the positive equilibrium. In Section 4, we consider the global existence of bifurcating periodic solutions. In Section 5, we will give some numerical simulations to support the theoretical prediction. In Section 6, a brief discussion is given.

## 2. Positivity and Boundedness

In this section, we study the positivity and boundedness of solutions of system (5) with initial condition (6).

**Theorem 1**

Solutions of system (5) with initial condition (6) are positive for all *t*⩾0.

**Proof**

Assume (*S*(*t*), *I*(*t*), *R*(*t*)) is a solution of system (5) with initial condition (6). Let us consider *I*(*t*) for *t*⩾0. It follows from the second equation of system (5) that

(7) $$\begin{array}{c} I\left(t\right)=I\left(0\right)e^{\int_{0}^{t}((\beta_{1}-(\left(\beta_{2}I(s-\tau )\right)/\left(m+I(s-\tau )\right)))S(s)-(d+\mu +\delta ))ds}. \end{array}$$

From the initial condition (6), we have *I*(*t*) > 0, for *t*⩾0. Then, from the third equation of system (5), we have

(8) $$\begin{array}{c} \frac{dR\left(t\right)}{dt}=\mu I\left(t\right)-\left(d+\gamma \right)R\left(t\right)>-\left(d+\gamma \right)R\left(t\right). \end{array}$$

A comparison argument shows that

(9) $$\begin{array}{c} R\left(t\right)⩾R\left(0\right)e^{\int_{0}^{t}(-(d+\gamma ))ds}. \end{array}$$

From the initial condition (6), we have *R*(*t*) > 0, for *t*⩾0.

Next, we prove that *S*(*t*) is positive. Assume the contrary; then, let *t*_1_ be the first time such that *S*(*t*_1_) = 0. By the first equation of (5) we have

(10) $$\begin{array}{c} {\frac{dS(t)}{dt}|}_{t=t_{1}}=b+\gamma R\left(t_{1}\right)>0. \end{array}$$

This means *S*(*t*) < 0 for *t* ∈ (*t*_1_ − *ɛ*, *t*_1_), where *ɛ* is an arbitrarily small positive constant. This leads to a contradiction. It follows that *S*(*t*) is always positive for *t*⩾0. This ends the proof.

**Theorem 2**

Solutions of system (5) with initial condition (6) are ultimately bounded.

**Proof**

From Theorem 1, solutions of system (5) with initial condition (6) are positive for all *t*⩾0. Let *N*(*t*) = *S*(*t*) + *I*(*t*) + *R*(*t*). From (5), we have

(11) $$\begin{array}{c} \frac{dN\left(t\right)}{dt}=b-dN\left(t\right)-\delta I\left(t\right)<b-dN\left(t\right). \end{array}$$

Therefore, *N*(*t*)<(*b*/*d*) + *ɛ* for all large *t*, where *ɛ* is an arbitrarily small positive constant. Thus, *S*(*t*), *I*(*t*), and *R*(*t*) are ultimately bounded.

## 3. Local Stability and Hopf Bifurcation Analysis

### 3.1. Previous Results

We now state some key results from [17], which provide the context for the main results of this paper. The basic reproduction number [17, 21] for the model is

(12) $$\begin{array}{c} R_{0}=\frac{b\beta_{1}}{d\left(d+\mu +\delta \right)}. \end{array}$$

From [21], when *τ* = 0, system (5) has a disease-free equilibrium *E*_0_ = (*b*/*d*, 0,0), which exists for all parameter values. When *R*_0_ > 1, system (5) has a unique endemic equilibrium E* = (S*, I*, R*), where

(13) $$\begin{array}{c} S^{*}=\frac{\left(d+\mu +\gamma \right)\left(m+I^{*}\right)}{\beta_{1}\left(m+I^{*}\right)-\beta_{2}I^{*}},\\ R^{*}=\frac{\mu I^{*}}{d+\gamma },\\ H_{1}I^{*2}+H_{2}I^{*}+H_{3}=0,\\ H_{1}=-\frac{1}{d+\gamma }\left(\beta_{1}-\beta_{2}\right)\left[\gamma \left(d+\delta \right)+d\left(d+\mu +\delta \right)\right],\\ H_{2}=-\frac{d\beta_{1}m\mu }{d+\gamma }-\beta_{1}m\left(d+\delta \right)-b\beta_{2}+b\beta_{1}\left(1-\frac{1}{R_{0}}\right),\\ H_{3}=dm\left(d+\mu +\delta \right)\left(R_{0}-1\right). \end{array}$$

Denoting Γ = {(*S*, *I*, *R*) ∈ *ℛ*_+_^3^ | 0 < *S* + *I* + *R* ⩽ *b*/*d*}, the following results in [17, 21] are here just recalled.

**Lemma 3**

For *τ* = 0, we have the following.

- The disease-free equilibrium *E*_0_ is globally asymptotically stable if *R*_0_ < 1 and unstable if *R*_0_ > 1 in the set Γ.
- The endemic equilibrium E* is globally asymptotically stable if *R*_0_ > 1 in the set Γ.

### 3.2. Local Stability at *E*_0_

The characteristic equation of system (5) at *E*_0_ is

(14) $$\begin{array}{c} \det\left[\begin{array}{ccc} \lambda +d & \frac{\beta_{1}b}{d} & -\gamma \\ 0 & \lambda +\left(d+\mu +\delta -\frac{\beta_{1}b}{d}\right) & 0\\ 0 & -\mu & \lambda +\left(d+\gamma \right) \end{array}\right]=0, \end{array}$$

which is equivalent to

(15) $$\begin{array}{c} \left(\lambda +d\right)\left(\lambda +d+\gamma \right)\left(\lambda +d+\mu +\delta -\frac{\beta_{1}b}{d}\right)=0. \end{array}$$

It is easy to see that, when *R*_0_ < 1, (15) has three negative roots and that, when *R*_0_ > 1, (15) has one positive root and two negative roots. Thus, we have the following.

**Theorem 4**

For any time delay *τ*⩾0, we have the following:

- the disease-free equilibrium *E*_0_ is locally asymptotically stable if *R*_0_ < 1.
- the disease-free equilibrium *E*_0_ is unstable if *R*_0_ > 1.

### 3.3. Local Stability and Hopf Bifurcation at E*

In this subsection, we suppose that *R*_0_ > 1. In what follows, using time delay as the bifurcation parameter, we investigate the Hopf bifurcation for system (5) and the stability of E* by using the method in [25, 26].

The characteristic equation of system (5) at E* is

(16) $$\begin{array}{c} \det\left[\begin{array}{ccc} \lambda -a_{1} & -a_{2}-a_{6}e^{-\lambda \tau } & -\gamma \\ -a_{3} & \lambda -a_{4}+a_{6}e^{-\lambda \tau } & 0\\ 0 & -\mu & \lambda -a_{5} \end{array}\right]=0, \end{array}$$

where *a*_1_ = −*d* − *β*_1_ I* + (*β*_2_ *I* ^∗2^/(*m* + I*)), *a*_2_ = −*β*_1_ S* + (*β*_2_ S*I*/(*m* + I*)), *a*_3_ = *β*_1_ I* − (*β*_2_ *I* ^∗2^/(*m* + I*)), *a*_4_ = −(*d* + *μ* + *β*) + *β*_1_ S* − (*β*_2_ S*I*/(*m* + I*)), *a*_5_ = −(*d* + *γ*), and *a*_6_ = (*mβ*_2_ S*I*/(*m* + I*)^2^). Equation (16) is equivalent to

(17) $$\begin{array}{c} \lambda^{3}+b_{1}\lambda^{2}+b_{2}\lambda +b_{3}+\left(b_{4}\lambda^{2}+b_{5}\lambda +b_{6}\right)e^{-\lambda \tau }=0, \end{array}$$

where *b*_1_ = −(*a*_1_ + *a*_4_ + *a*_5_), *b*_2_ = *a*_1_ *a*_4_ + *a*_1_ *a*_5_ + *a*_4_ *a*_5_ − *a*_2_ *a*_3_, *b*_3_ = −*a*_1_ *a*_4_ *a*_5_ + *a*_2_ *a*_3_ *a*_5_ − *a*_3_ *γμ*, *b*_4_ = *a*_6_, *b*_5_ = −*a*_6_(*a*_1_ + *a*_3_ + *a*_5_), and *b*_6_ = *a*_5_ *a*_6_(*a*_1_ + *a*_3_).

Obviously, *iω* is a root of (17) if and only if *ω* satisfies

(18) −ω3i−b1ω2+b2ωi+b3  +(−b4ω2+b5ωi+b6)(cos⁡ωτ−isinωτ)=0.

Separating the real and imaginary parts, we have

(19) $$\begin{array}{c} b_{1}\omega^{2}-b_{3}=\left(b_{6}-b_{4}\omega^{2}\right)\cos\omega \tau +b_{5}\omega \sin\omega \tau ,\\ -\omega^{3}+b_{2}\omega =\left(b_{6}-b_{4}\omega^{2}\right)\sin\omega \tau -b_{5}\omega \cos\omega \tau , \end{array}$$

which is equivalent to

(20) ω6+(b12−b42−2b2)ω4  +(b22−2b1b3−b52+2b4b6)ω2+b32−b62=0.

Let *z* = *ω* ^2^ and denote *p* = *b*_1_^2^ − *b*_4_^2^ − 2*b*_2_, *q* = *b*_2_^2^ − 2*b*_1_ *b*_3_ − *b*_5_^2^ + 2*b*_4_ *b*_6_, and *r* = *b*_3_^2^ − *b*_6_^2^. Then (20) becomes

(21) $$\begin{array}{c} z^{3}+pz^{2}+qz+r=0. \end{array}$$

Next, we need to seek the conditions under which (21) has at least one positive root. Denote

(22) $$\begin{array}{c} h\left(z\right)=z^{3}+pz^{2}+qz+r. \end{array}$$

Since lim⁡_*z*→+*∞*_⁡*h*(*z*) = +*∞*, we conclude that if *r* < 0, then (21) has at least one positive root.

From (22), we have

(23) $$\begin{array}{c} h^{\prime }\left(z\right)=3z^{2}+2pz+q. \end{array}$$

Clearly, if Δ = *p* ^2^ − 3*q* ⩽ 0, then the function *h*(*z*) is monotone increasing in *z* ∈ [0, +*∞*). Thus, when *r*⩾0 and Δ ⩽ 0, (21) has no positive real root. On the other hand, when *r*⩾0 and Δ > 0, the following equation

(24) $$\begin{array}{c} 3z^{2}+2pz+q=0 \end{array}$$

has two real roots

(25) $$\begin{array}{c} z_{1}^{*}=\frac{-p+\sqrt{\Delta }}{3},\;\;z_{2}^{*}=\frac{-p-\sqrt{\Delta }}{3}. \end{array}$$

It is easy to see that $h^{\prime \prime }(z_{1}^{*})=2\sqrt{\Delta }>0$ and $h^{\prime \prime }(z_{2}^{*})=-2\sqrt{\Delta }<0$. It follows that *z*_1_* and *z*_2_* are the local minimum and the local maximum of *h*(*z*), respectively. Hence, we have the following simple property.

**Lemma 5**

Suppose that *r*⩾0 and Δ > 0. Then (21) has positive root if and only if *z*_1_* > 0 and *h*(*z*_1_*) ⩽ 0.

From Lemma 5 and the discussion above, we have the following.

**Lemma 6**

For the polynomial equation (21), we have the following results.

- If *r* < 0, then (21) has at least one positive root.
- If *r*⩾0 and Δ = *p* ^2^ − 3*q* ⩽ 0, then (21) has no positive root.
- If *r*⩾0 and Δ = *p* ^2^ − 3*q* > 0, then (21) has positive roots if and only if $z_{1}^{*}=(\left(-p+\sqrt{\Delta }\right)/3)>0$ and *h*(*z*_1_*) ⩽ 0.

Suppose that (21) has positive root. Without loss of generality, we assume that it has three positive roots, defined by *z*_1_, *z*_2_, and *z*_3_, respectively. Then (20) has three positive roots

(26) $$\begin{array}{c} \omega_{1}=\sqrt{z_{1}},\;\;\omega_{2}=\sqrt{z_{2}},\;\;\omega_{3}=\sqrt{z_{3}}. \end{array}$$

From (19), we have

(27) $$\begin{array}{c} \cos\omega \tau =\frac{b_{5}\omega^{2}\left(\omega^{2}-b_{2}\right)-\left(b_{1}\omega^{2}-b_{3}\right)\left(b_{4}\omega^{2}-b_{6}\right)}{{\left(b_{4}\omega^{2}-b_{6}\right)}^{2}+b_{1}^{2}\omega^{2}}. \end{array}$$

Thus, if we denote

(28) τk(j)=1ωk{cos⁡−1(b5ωk2(ωk2−b2)−(b1ωk2−b3)(b4ωk2−b6)(b4ωk2−b6)2+b12ωk2)+2jπ},

where *k* = 1,2, 3 and *j* = 0,1, 2,…, then ±*iω*_*k*_ is a pair of purely imaginary roots of (17) with *τ* = *τ*_*k*_^(*j*)^. Define

(29) $$\begin{array}{c} \tau_{0}=\tau_{k_{0}}^{\left(0\right)}=\underset{k\in \{1,2,3\}}{\min}\left\{\tau_{k}^{\left(0\right)}\right\},\;\;\omega_{0}=\omega_{k_{0}}. \end{array}$$

Note that, from Lemma 3, when *τ* = 0, the endemic equilibrium E* is stable if *R*_0_ > 1. Till now, we can employ a result from Ruan and Wei [25] to analyze (17), which is stated as follows.

**Lemma 7**

Consider the exponential polynomial

(30) P(λ,e−λτ1,…,e−λτm)  =λn+p1(0)λn−1+⋯+pn−1(0)λ+pn(0)   +(p1(1)λn−1+⋯+pn−1(1)λ+pn(1))e−λτ1   +⋯+(p1(m)λn−1+⋯+pn−1(m)λ+pn(m))e−λτm,

where *τ*_*i*_⩾0  (*i* = 1,2,…, *m*) and *p*_*j*_^(*i*)^  (*i* = 0,1,…, *m*; *j* = 1,2,…, *n*) are constants. As (*τ*_1_, *τ*_2_,…, *τ*_*m*_) vary, the sum of the order of the zeros of *P*  (*λ*, *e* ^−*λτ*_1_^,…, *e* ^−*λτ*_*m*_^) on the open right half plane can change only if a zero appears on or crosses the imaginary axis.

Applying Lemmas 6 and 7 and the discussion above, we obtain the following lemma.

**Lemma 8**

For the third degree transcendental equation (17), we have the following:

- if *r*⩾0 and Δ = *p* ^2^ − 3*q* ⩽ 0, then all roots of (17) have negative real parts for all *τ*⩾0;
- if either *r* < 0 or *r*⩾0, Δ = *p* ^2^ − 3*q* > 0, $z_{1}^{*}=(\left(-p+\sqrt{\Delta }\right)/3)>0$, and *h*(*z*_1_*) ⩽ 0, then all roots of (17) have negative real parts for *τ* ∈ [0, *τ*_0_).

Let

(31) $$\begin{array}{c} \lambda \left(\tau \right)=\alpha \left(\tau \right)+i\omega \left(\tau \right) \end{array}$$

be the root of (17) near *τ* = *τ*_*k*_^(*j*)^ satisfying *α*(*τ*_*k*_^(*j*)^) = 0 and *ω*(*τ*_*k*_^(*j*)^) = *ω*_*k*_. Then, from Lemma  8 in [26], we have the following transversality condition.

**Lemma 9**

Suppose that *z*_*k*_ = *ω*_*k*_^2^ and *h*′(*z*_*k*_) ≠ 0, where *h*(*z*) is defined by (22). Then

(32) d(Reλ(τk(j)))dτ≠0,

and *d*(*Reλ*(*τ*_*k*_^(*j*)^))/*dτ* has the same sign with *h*′(*z*_*k*_).

The proof of Lemma 9 is similar to that in the proof of Lemma  8 in [26], and here we omit it.

Then, from the above discussion and Lemmas 8 and 9, we have the following theorem.

**Theorem 10**

Suppose *R*_0_ > 1 holds, and *τ*_*k*_^(*j*)^, *ω*_0_, and *τ*_0_ are defined by (28) and (29), respectively. Then

- if *r*⩾0 and Δ = *p* ^2^ − 3*q* ⩽ 0, the endemic equilibrium E* of system (5) is locally asymptotically stable for all *τ*⩾0;
- if either *r* < 0 or *r*⩾0, Δ = *p* ^2^ − 3*q* > 0, $z_{1}^{*}=(\left(-p+\sqrt{\Delta }\right)/3)>0$, and *h*(*z*_1_*) ⩽ 0, the endemic equilibrium E* of system (5) is locally asymptotically stable for *τ* ∈ [0, *τ*_0_);
- if the conditions of (ii) are satisfied and *h*′(*z*_*k*_) ≠ 0, then system (5) exhibits Hopf bifurcation at the endemic equilibrium E* when *τ* pass through *τ* = *τ*_*k*_^(*j*)^.

## 4. Global Continuation of Local Hopf Bifurcations

In this section, we study the global continuation of periodic solutions bifurcating from the positive equilibrium E* of system (5).

Throughout this section, we follow closely the notations in [27]. For simplification of notations, setting *z*(*t*) = (*z*_1_(*t*), *z*_2_(*t*), *z*_3_(*t*))^*T*^ = (*S*(*t*), *I*(*t*), *R*(*t*))^*T*^, we may rewrite system (5) as the following functional differential equation:

(33) $$\begin{array}{c} \dot{z}\left(t\right)=ℱ\left(z_{t},\tau ,p\right), \end{array}$$

where *z*_*t*_(*θ*) = (*z*_1*t*_(*θ*), *z*_2*t*_(*θ*), *z*_3*t*_(*θ*))^*T*^ = (*z*_1_(*t* + *θ*), *z*_2_(*t* + *θ*), *z*_3_(*t* + *θ*))^*T*^ ∈ *𝒞*([−*τ*, 0], **R** ^3^). It is obvious that if *R*_0_ > 1 holds, then system (5) has a semitrivial equilibrium *E*_0_(*b*/*d*, 0,0) and a positive equilibrium E*(S*, I*, R*). Following the work of [27], we need to define

(34) X=𝒞([−τ,0],R2),Γ=Cl{(z,τ,p)∈X×R×R+;z  is  a  nonconstant  periodic  solution  of  (33)},𝒩={(z¯,τ¯,p¯);ℱ(z¯,τ¯,p¯)=0}.

Let *ℓ*_(E*,*τ*_*j*_,2*π*/*ω*_0_)_ denote the connected component passing through (E*, *τ*_*j*_, 2*π*/*ω*_0_) in Γ, where *τ*_*j*_ and *ω*_0_ are defined by (28) and (29). From Theorem 10, we know that *ℓ*_(E*,*τ*_*j*_,2*π*/*ω*_0_)_ is nonempty.

We first state the global Hopf bifurcation theory due to Wu [27] for functional differential equations.

**Lemma 11**

Assume that (*z*_∗_, *τ*, *p*) is an isolated center satisfying the hypotheses (*A*_1_)–(*A*_4_) in [27]. Denote by *ℓ*_(*z*_∗_,*τ*,*p*)_ the connected component of (*z*_∗_, *τ*, *p*) in Γ. Then either

(i) *ℓ*_(*z*_∗_,*τ*,*p*)_ is unbounded, or
(ii) *ℓ*_(*z*_∗_,*τ*,*p*)_ is bounded, *ℓ*_(*z*_∗_,*τ*,*p*)_∩Γ is finite, and
  (35) $$\begin{array}{c} \sum_{(z,\tau ,p)\in \ell_{\left(z_{*},\tau ,p\right)}\cap 𝒩}\gamma_{m}\left(z_{*},\tau ,p\right)=0, \end{array}$$

for all *m* = 1,2,…, where *γ*_*m*_(*z*_∗_, *τ*, *p*) is the *m*th crossing number of (*z*_∗_, *τ*, *p*) if *m* ∈ *J*(*z*_∗_, *τ*, *p*), or it is zero if otherwise.

Clearly, if (ii) in Lemma 11 is not true, then *ℓ*_(*z*_∗_,*τ*,*p*)_ is unbounded. Thus, if the projections of *ℓ*_(*z*_∗_,*τ*,*p*)_ onto *z*-space and onto *p*-space are bounded, then the projection onto *τ*-space is unbounded. Further, if we can show that the projection of *ℓ*_(*z*_∗_,*τ*,*p*)_ onto *τ*-space is away from zero, then the projection of *ℓ*_(*z*_∗_,*τ*,*p*)_ onto *τ*-space must include interval [*τ*, +*∞*). Following this ideal, we can prove our results on the global continuation of local Hopf bifurcation.

From Theorems 1 and 2, it is easy to have the following.

**Lemma 12**

If the condition *R*_0_ > 1 holds, then all nonconstant periodic solutions of (5) with initial condition (6) are uniformly bounded.

From [21], we know the following lemma.

**Lemma 13**

If the condition *R*_0_ > 1 holds, then when *τ* = 0, the positive equilibrium E* is globally stable in **R**_+_^3^.

**Lemma 14**

If *R*_0_ > 1, then system (5) has no nonconstant periodic solution with period *τ*.

**Proof**

Suppose for a contradiction that system (5) has nonconstant periodic solution with period *τ*. Then the following system of ordinary differential equations has nonconstant periodic solution:

(36) $$\begin{array}{c} \frac{dS\left(t\right)}{dt}=b-dS\left(t\right)-\left(\beta_{1}-\frac{\beta_{2}I\left(t\right)}{m+I\left(t\right)}\right)S\left(t\right)I\left(t\right)+\gamma R\left(t\right),\\ \frac{dI\left(t\right)}{dt}=\left(\beta_{1}-\frac{\beta_{2}I\left(t\right)}{m+I\left(t\right)}\right)S\left(t\right)I\left(t\right)-\left(d+\mu +\delta \right)I\left(t\right),\\ \frac{dR\left(t\right)}{dt}=\mu I\left(t\right)-\left(d+\gamma \right)R\left(t\right). \end{array}$$

System (36) has the same equilibria as system (5), that is, *E*_0_(Λ/*d*, 0,0) and a positive equilibrium E*(S*, I*, R*). Note that *I*-axis and *R*-axis are the invariable manifold of system (36) and the orbits of system (36) do not intersect each other. Thus, there is no solution that crosses the coordinate axis. On the other hand, note the fact that if system (36) has a periodic solution, then there must be the equilibrium in its interior and *E*_0_ are located on the coordinate axis. Thus, we conclude that the periodic orbit of system (36) must lie in the first quadrant. From Lemma 13, the positive equilibrium is asymptotically stable and globally stable in **R**_+_^3^; thus, there is no periodic orbit in the first quadrant. This ends the proof.

**Theorem 15**

Let *ω*_0_ and *τ*_*j*_  (*j* = 0,1,…) be defined in (28) and (29). If *R*_0_ > 1, then system (5) has at least *j* − 1 periodic solutions for every *τ* > *τ*_*j*_  (*j* = 1,2,…).

**Proof**

It is sufficient to prove that the projection of *ℓ*_(E*,*τ*_*j*_,2*π*/*ω*_0_)_ onto *τ*-space is $\left[\bar{\tau },+\infty \right)$ for each *j* > 0, where $\bar{\tau }\leq \tau_{j}$.

The characteristic matrix of (33) at an equilibrium $\bar{z}=({\bar{z}}^{\left(1\right)},{\bar{z}}^{\left(2\right)},{\bar{z}}^{\left(3\right)})\in R^{3}$ takes the following form:

(37) $$\begin{array}{c} \Delta \left(\bar{z},\tau ,p\right)\left(\lambda \right)=\lambda Id-Dℱ\left(\bar{z},\bar{\tau },\bar{p}\right)\left(e^{\lambda }Id\right), \end{array}$$

where $\left(\bar{z},\bar{\tau },\bar{p}\right)$ is called a center if $ℱ(\bar{z},\bar{\tau },\bar{p})=0$ and $\det(\Delta (\bar{z},\bar{\tau },\bar{p})((2\pi /p)i))=0$. A center is said to be isolated if it is the only center in some neighborhood of $\left(\bar{z},\bar{\tau },\bar{p}\right)$. It follows from (37) that

(38) det⁡(Δ(E0,τ,p)(λ)) =(λ+d)(λ+d+γ)(λ+d+μ+δ−β1bd)=0,det⁡(Δ(E∗,τ,p)(λ)) =λ3+b1λ2+b2λ+b3+(b4λ2+b5λ+b6)e−λτ=0,

where *b*_1_, *b*_2_, *b*_3_, *b*_4_, *b*_5_, and*b*_6_ are defined as in Section 3. From the discussion in Section 3, each of (38) has no purely imaginary root provided that *R*_0_ < 1. Thus, we conclude that (33) has no center of the form as (*E*_0_, *τ*, *p*) and (E*, *τ*, *p*). On the other hand, from the discussion in Section 3 about the local Hopf bifurcation, it is easy to verify that (E*, *τ*_*j*_, 2*π*/*ω*_0_) is an isolated center, and there exist *ϵ* > 0, *δ* > 0, and a smooth curve *λ* :  (*τ*_*j*_ − *δ*, *τ*_*j*_ + *δ*) → *𝒞* such that det⁡(Δ(*λ*(*τ*))) = 0, |*λ*(*τ*) − *ω*_0_| < *ϵ* for all *τ* ∈ [*τ*_*j*_ − *δ*, *τ*_*j*_ + *δ*] and

(39) λ(τj)=ω0i,  dReλ(τ)dτ|τ=τj>0.

Let

(40) $$\begin{array}{c} \Omega_{\epsilon ,2\pi /\omega_{0}}=\left\{\left(\eta ,p\right);\;0<\eta <\epsilon ,\;\left|p-\frac{2\pi }{\omega_{0}}\right|<\epsilon \right\}. \end{array}$$

It is easy to verify that on [*τ*_*j*_ − *δ*, *τ*_*j*_ + *δ*]×∂*Ω*_*ϵ*,2*π*/*ω*_0__,

(41) det⁡⁡(Δ(E∗,τ,p)(η+2πpi))=0    iff  η=0,  τ=τj,  p=2πω0.

Therefore, the hypotheses (*A*_1_)–(*A*_4_) in [27] are satisfied. Moreover, if we define

(42) H±(E∗,τj,2πω0)(η,p)  =det⁡(Δ(E∗,τj±δ,p)(η+2πpi)),

then we have the crossing number of isolated center (E*, *τ*_*j*_, 2*π*/*ω*_0_) as follows:

(43) γ(E∗,τj,2πω0) =degB(H−(E∗,τj,2πω0),Ωϵ,2π/ω0)  −degB(H+(E∗,τj,2πω0),Ωϵ,2π/ω0)=−1.

Thus, we have

(44) $$\begin{array}{c} \sum_{(\bar{z},\bar{\tau },\bar{p})\in {𝒞}_{\left(E^{*},\tau_{j},2\pi /\omega_{0}\right)}}\gamma \left(\bar{z},\bar{\tau },\bar{p}\right)<0, \end{array}$$

where $\left(\bar{z},\bar{\tau },\bar{p}\right)$ has all or parts of the form (E*, *τ*_*k*_, 2*π*/*ω*_0_)  (*k* = 0,1,…).

It follows from Lemma 11 that the connected component *ℓ*_(E*,*τ*_*j*_,2*π*/*ω*_0_)_ through (E*, *τ*_*j*_, 2*π*/*ω*_0_) in Γ is unbounded. From (28), we can know that if *R*_0_ > 1 holds, for *j* ≥ 1,

(45) $$\begin{array}{c} \tau_{j}=\frac{1}{\omega_{0}}\left\{\cos^{-1}\left(\frac{b_{4}\omega^{2}-b_{2}b_{4}-b_{1}b_{3}\omega^{2}}{b_{4}^{2}+b_{3}^{2}\omega^{2}}\right)+2j\pi \right\}>\frac{2\pi }{\omega_{0}}. \end{array}$$

Now we prove that the projection of *ℓ*_(E*,*τ*_*j*_,2*π*/*ω*_0_)_ onto *τ*-space is $\left[\bar{\tau },+\infty \right)$, where $\bar{\tau }\leq \tau_{j}$. Clearly, it follows from the proof of Lemma 14 that system (5) with *τ* = 0 has no nontrivial periodic solution. Hence, the projection of *ℓ*_(E*,*τ*_*j*_,2*π*/*ω*_0_)_ onto *τ*-space is away from zero.

For a contradiction, we suppose that the projection of *ℓ*_(E*,*τ*_*j*_,2*π*/*ω*_0_)_ onto *τ*-space is bounded; this means that the projection of *ℓ*_(E*,*τ*_*j*_,2*π*/*ω*_0_)_ onto *τ*-space is included in an interval (0, τ*). Noticing 2*π*/*ω*_0_ < *τ*_*j*_ and applying Lemma 14, we have 0 < *p* < τ* for (*z*(*t*), *τ*, *p*) belonging to *ℓ*_(E*,*τ*_*j*_,2*π*/*ω*_0_)_. Applying Lemma 12, we know that the projection of *ℓ*_(E*,*τ*_*j*_,2*π*/*ω*_0_)_ onto *z*-space is bounded. So the component of *ℓ*_(E*,*τ*_*j*_,2*π*/*ω*_0_)_ is bounded. It contradicts our conclusion that *ℓ*_(E*,*τ*_*j*_,2*π*/*ω*_0_)_ is unbounded. The contradiction implies that the projection of *ℓ*_(E*,*τ*_*j*_,2*π*/*ω*_0_)_ onto *τ*-space is unbounded above.

Hence, system (5) has at least *j* − 1 periodic solution for every *τ* > *τ*_*j*_, (*j* = 1,2,…). This completes the proof.

## 5. Numerical Simulation

**Example 1**

In this case, we set *b* = 10, *d* = 0.02, *β*_1_ = 0.0002, *β*_2_ = 0.00018, *m* = 30, *δ* = 0.1, *μ* = 0.05, and *γ* = 0.01. From (12), we compute *R*_0_ = 0.5882 < 1. Furthermore, from (13), system (5) has only a disease-free equilibrium *E*_0_ = (500,0, 0). From Theorem 4, we know that the disease-free equilibrium *E*_0_ is locally asymptotically stable for any time delay *τ*⩾0.

**Figure 1:**
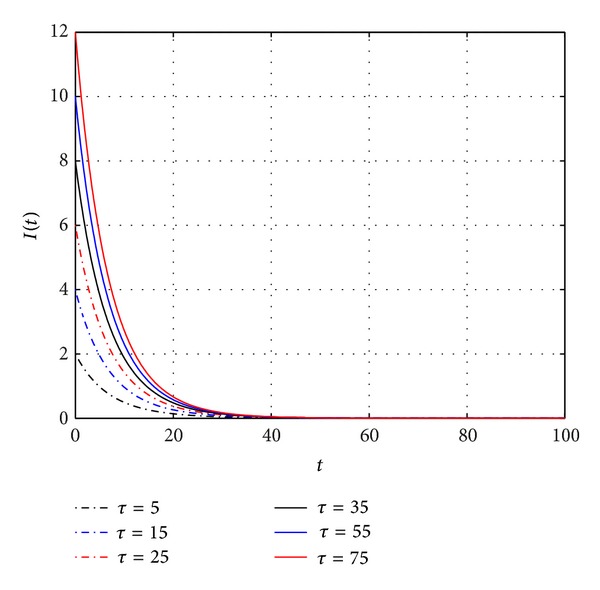
The trajectories of *I*(*t*) with *τ* = 5, 15, 25, 35, 45, 55, respectively. *E*_0_ is always stable.

Figure 1 shows that *E*_0_ is locally asymptotically stable, and the trajectories of *I*(*t*) always converge to zero for *τ* taking some different values.

**Example 2**

In this case, we set *b* = 10, *d* = 0.02, *β*_1_ = 0.002, *β*_2_ = 0.0018, *m* = 30, *δ* = 0.1, *μ* = 0.05, and *γ* = 0.01. From (12), we compute *R*_0_ = 5.8824 > 1. Furthermore, from (13), we get a disease-free equilibrium *E*_0_ = (500,0, 0) and an endemic equilibrium E* = (178.7543,41.9016,69.8360) of system (5). From the algorithm of Section 3.3, we can compute *τ*_0_ ≈ 20.4343 and *h*′(*z*_*k*_) = 13.2438 > 0. Thus, from Theorems 4 and 10, we know that the disease-free equilibrium *E*_0_ is unstable for all *τ*⩾0 and that the endemic equilibrium E* is stable for *τ* ∈ [0,20.4343). When *τ* crosses *τ*_0_, a family of periodic orbits bifurcate from E*.

**Figure 2:**
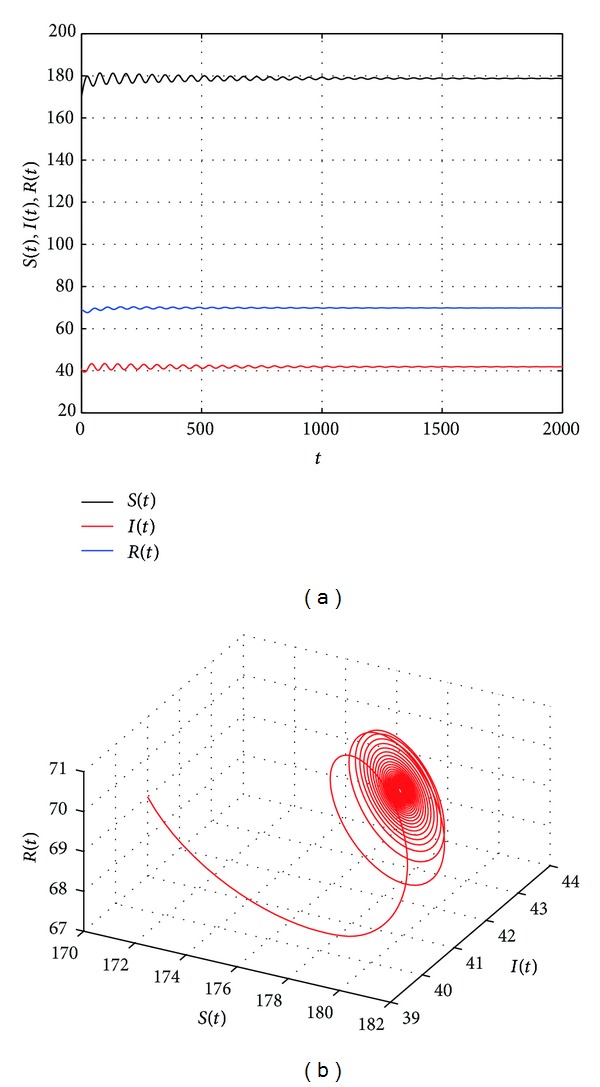
The trajectories and phase graphs of system (5) with *τ* = 19. E* is stable.

**Figure 3:**
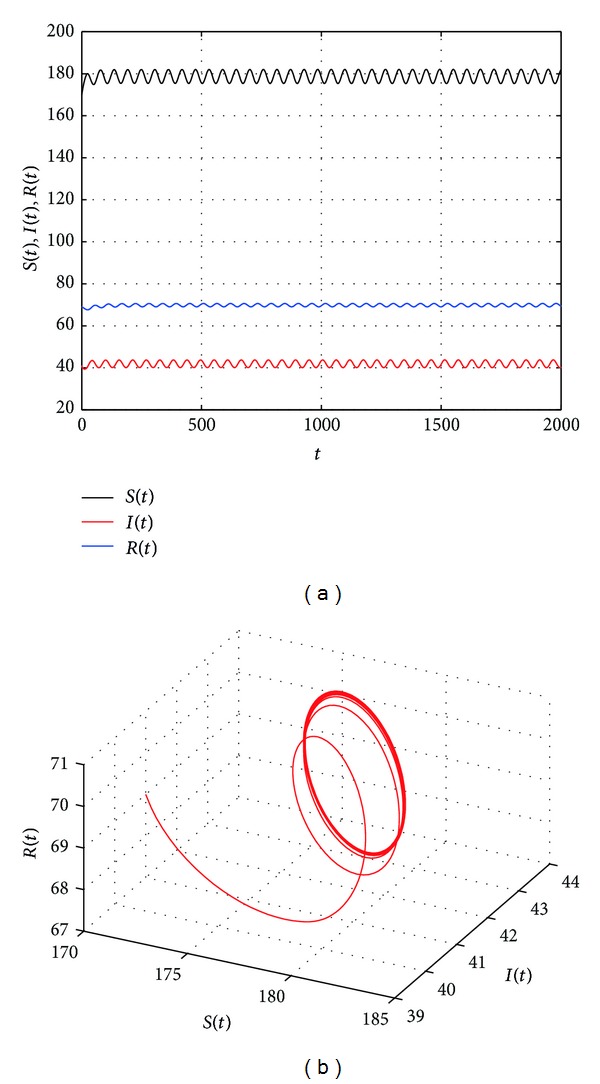
The trajectories and phase graphs of system (5) with *τ* = 20.5, E* is unstable and a periodic orbit bifurcate from E*.

**Figure 4:**
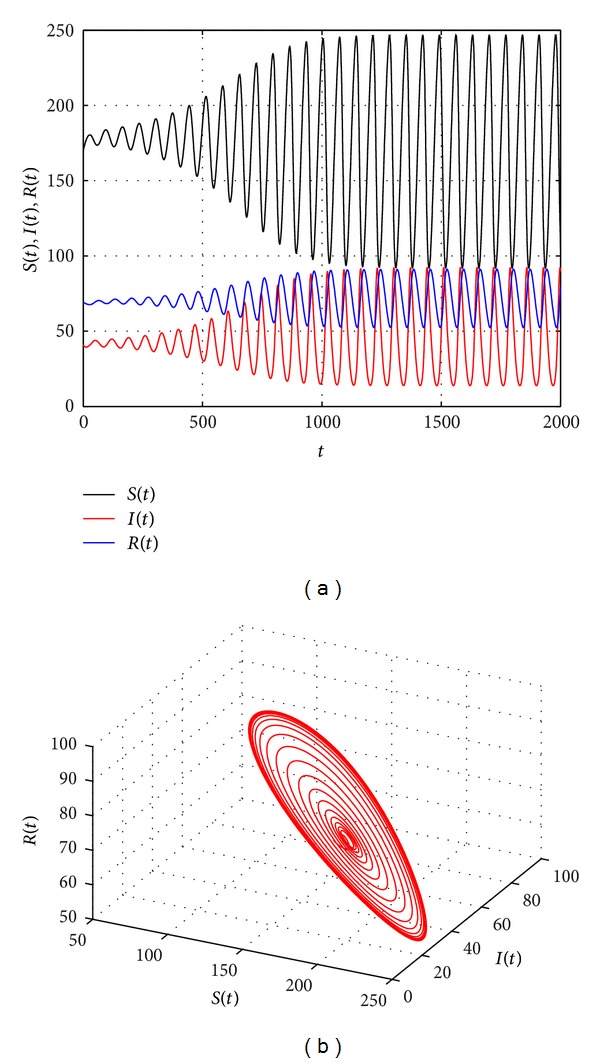
The trajectories and phase graphs of system (5) with *τ* = 30. E* is unstable and a periodic orbit bifurcate from E*.

**Figure 5:**
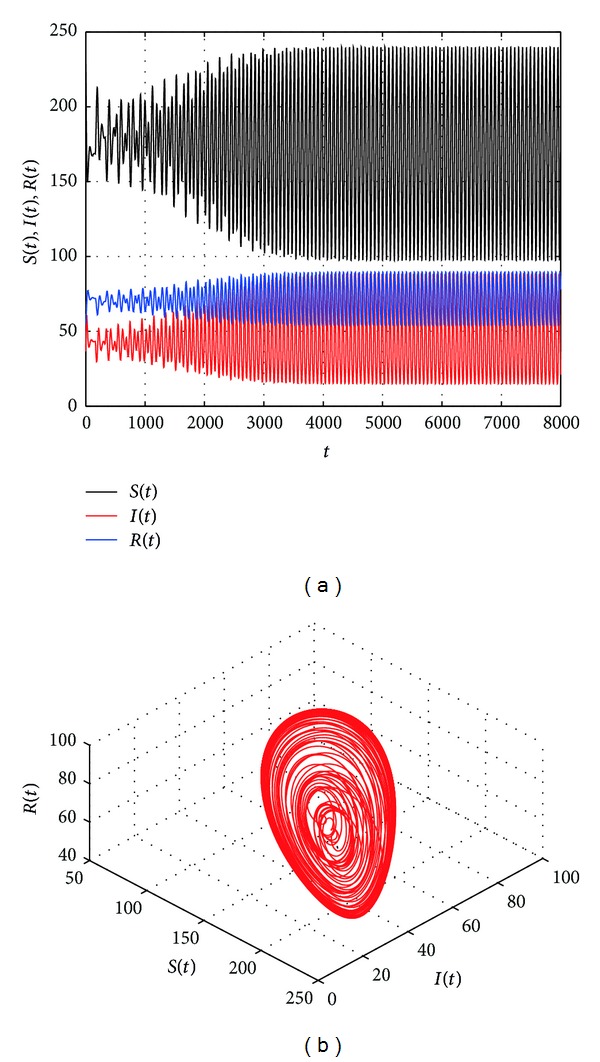
The trajectories and phase graphs of system (5) with *τ* = 160. E* is unstable and a periodic orbit bifurcate from E*.

Figure 2 shows that the endemic equilibrium E* is stable with *τ* = 19.  Figure 3 shows that the endemic equilibrium E* is unstable and a periodic orbit bifurcate from E* with *τ* = 20.5.  Figure 4 shows that the endemic equilibrium E* is still unstable and a periodic orbit bifurcate from E* with *τ* = 30. We can see from Figures 3 and 4 that the period and amplitude of the oscillation are increasing with the increasing of time delay. Furthermore, Figure 5 shows that the local Hopf bifurcation implies the global Hopf bifurcation after the second critical value of *τ*_1_ ≈ 146.4764.

## 6. Discussion

In this paper, we proposed an SIRS epidemic model incorporating media coverage with time delay. We first investigated the positivity and boundedness of the solution of system (5) and show that the solution of system (5) with the initial condition (6) is positive and bounded.

Secondly, we studied the stability of the disease-free equilibrium. Our results show that the disease-free equilibrium is globally stable for all *τ*⩾0 when the basic reproduction number *R*_0_ < 1. This is to say, the time delay in media coverage cannot influence the stability of the disease-free equilibrium. In other words, we can ignore the effect of time delay for *R*_0_ < 1.

However, when *R*_0_ > 1, the stability of the endemic equilibrium will be affected by the time delay in media coverage. We found that there existed a critical value of time delay *τ*, such that the stability of the endemic equilibrium changed and periodic oscillations occurred when the time delay passes through this critical value. Furthermore, we show that the local Hopf bifurcation implies the global Hopf bifurcation after the second critical value of delay.

These results mean that, when *R*_0_ > 1 and the time delay is small enough, the epidemic will eventually become endemic disease. However, if the delay of information about and appraisal of an epidemic on media coverage is too large, it will lead to repeated episodes of epidemic, and then it is unfavourable for the containment of the epidemic. We suggest that it is helpful for controlling epidemic to communicate about the outbreak of an epidemic as soon as possible.
